# Recognition of Cognitive Impairment in Adult Moyamoya Disease: A Classifier Based on High-Order Resting-State Functional Connectivity Network

**DOI:** 10.3389/fncir.2020.603208

**Published:** 2020-12-21

**Authors:** Yu Lei, Xi Chen, Jia-Bin Su, Xin Zhang, Heng Yang, Xin-Jie Gao, Wei Ni, Liang Chen, Jin-Hua Yu, Yu-Xiang Gu, Ying Mao

**Affiliations:** ^1^Department of Neurosurgery, Huashan Hospital, Fudan University, Shanghai, China; ^2^Department of Electronic Engineering, Fudan University, Shanghai, China

**Keywords:** moyamoya disease, resting-state, fMRI, vascular cognitive impairment, functional connectivity, sliding window, functional dynamics

## Abstract

**Objective:** Vascular cognitive impairment (VCI) is a common complication in adult patients with moyamoya disease (MMD), and is reversible by surgical revascularization in its early stage of mild VCI. However, accurate diagnosis of mild VCI is difficult based on neuropsychological examination alone. This study proposed a method of dynamic resting-state functional connectivity (FC) network to recognize global cognitive impairment in MMD.

**Methods:** For MMD, 36 patients with VCI and 43 patients with intact cognition (Non-VCI) were included, as well as 26 normal controls (NCs). Using resting-state fMRI, dynamic low-order FC networks were first constructed with multiple brain regions which were generated through a sliding window approach and correlated in temporal dimension. In order to obtain more information of network interactions along the time, high-order FC networks were established by calculating correlations among each pair of brain regions. Afterwards, a sparse representation-based classifier was constructed to recognize MMD (experiment 1) and its cognitive impairment (experiment 2) with features extracted from both low- and high-order FC networks. Finally, the ten-fold cross-validation strategy was proposed to train and validate the performance of the classifier.

**Results:** The three groups did not differ significantly in demographic features (*p* > 0.05), while the VCI group exhibited the lowest MMSE scores (*p* = 0.001). The Non-VCI and NCs groups did not differ significantly in MMSE scores (*p* = 0.054). As for the classification between MMD and NCs, the area under the receiver operating characteristic curve (AUC), accuracy, sensitivity, and specificity of the classifier reached 90.70, 88.57, 93.67, and 73.08%, respectively. While for the classification between VCI and Non-VCI, the AUC, accuracy, sensitivity, and specificity of the classifier reached 91.02, 84.81, 80.56, and 88.37%, respectively.

**Conclusion:** This study not only develops a promising classifier to recognize VCI in adult MMD in its early stage, but also implies the significance of time-varying properties in dynamic FC networks.

## Introduction

Moyamoya disease (MMD) is a cerebrovascular disease characterized by both progressive stenosis of the terminal portion of the bilateral internal carotid arteries, and extensive network of cerebral collaterals (Suzuki and Kodama, 1983). Initial presentations of MMD are categorized into symptoms due to either cerebral ischemia (i.e., ischemic stroke) or compensatory mechanisms responding to the ischemia (i.e., intracranial bleeding from fragile collaterals) (Scott and Smith, 2009). Vascular cognitive impairment (VCI) is a common complication in adult patients with MMD, the diagnostic criteria of which is based on a link between the cognitive disorder and evidences of subclinical cerebral vascular damage or clinical stroke (Gorelick et al., 2011). The occurrence of VCI in MMD has been proved to be caused by subclinical cerebral vascular defects or clinical stroke (Karzmark et al., 2012). Furthermore, executive function is deemed to be predominantly impaired, and other cognitive domains of memory, language, and visuospatial functions may also be affected (Weinberg et al., 2011). As a continuous process, the VCI is reversible in its early stage of mild impairment through surgical revascularization (Gorelick et al., 2011; Lei et al., 2017a). Thus, the detection of mild or suspected VCI is considered as clinical significance. Neuropsychological assessment with cognitive tests is commonly used, but is limited to patients with disabilities, illiteracy, or uncooperativeness. Therefore, an alternative measurement with high sensitivity, reliability, and validity is needed.

In previous studies, the deterioration of intrinsic neural interaction has been proved to be the pathophysiological basis of VCI, and functional brain networks of executive-control, default-mode, and salience networks are found to be abnormal in adult MMD with VCI (Cocchi et al., 2014; Liang et al., 2016; Lei et al., 2017b). Furthermore, the executive-control network is deemed to be primarily deteriorated with disease progression (Schubert et al., 2014; Lei et al., 2017b). Normally, these resting-state fMRI studies are based on the setting that the blood oxygenation level dependent (BOLD) signal is stationary across the whole scanning session. This setting simplifies the computation and results in static functional connectivity (FC) patterns among brain regions. In detail, brain regions are commonly considered as vertexes and their functional interactions are regarded as edges for network construction.

However, the real neural synchronization is time-varying and shifts quickly to meet cognitive demands (Cole et al., 2013; Allen et al., 2014). These dynamic FC studies partition the entire time series of BOLD signal into numerous segments of subseries on the basis of sliding window approach, and a series of temporal FC networks are constructed for each segment of the signal (Wee et al., 2016). The adjacent networks are deemed to share a similar topological pattern and connection strength, and any changes can be recognized and utilized as discriminative information for VCI detection. In detail, the relative fixed positions of brain regions in each segment of the time series are deemed as vertexes, and their functional interactions between each pairs of regions are regarded as edges for a high-order FC network construction (Chen et al., 2016). The high-order FC networks can provide more neural interaction information and have been applied in several fMRI-based studies covering Alzheimer's disease (Chen et al., 2017) and Autism spectrum disorder (Zhao et al., 2018).

Recent studies reveal that sparse representation-based classification (SRC) outperforms some traditional classifiers like the support vector machine (Zhang et al., 2011; Yuan and Yan, 2012). The sparse representation coefficients are able to share some intrinsic relation among different features from one sample and such a classifier performs well in solving the problem of over-fitting (Cai et al., 2016). In this study, features from both low-order and high-order FC networks were extracted through this classifier, and trained to recognize MMD (experiment 1) and its cognitive impairment (experiment 2). Simply put, the aim was to develop an alternative to neuropsychological tests, a sensitive and reliable tool to recognize the general cognitive status of moyamoya patients.

## Materials and Methods

### Participants

A total of 79 patients with MMD and 26 matched healthy subjects as normal controls (NC) were enrolled in this study. Inclusion criteria has been published in a previous study of ours (Lei et al., 2020) and are detailed as follows: (i) right-handed Chinese patients aged over 18 years; (ii) physically capable of undergoing cognitive testing; (iii) no evidence of intracerebral hemorrhage and cortical or subcortical infarct larger than 8 mm in maximum dimension on structural images (Karzmark et al., 2012; Lei et al., 2014; Kazumata et al., 2015); (iv) no brain surgery before recruitment; (v) absence of any situation that could compromise cognition, such as diseases and drug use; and (vi) absence of severe systemic or other cerebrovascular diseases. Healthy young subjects with no memory complaints, mental diseases, any cerebrovascular disease were enrolled in the normal control group.

After assessed through the neuropsychological testing of global cognitive state using the mini-mental state examination (MMSE), 36 patients were diagnosed with VCI and the rest 43 patients were with intact cognition (Non-VCI) in accordance with 2011 AHA/ASA statement of VCI (Gorelick et al., 2011). In detail, the VCI is defined as 1.5 standard deviations below the mean score of the matched NC group. This study was approved by the Institutional Review Board in our hospital and was conducted in accordance with the Helsinki Declaration. All participants provided informed consent.

### Image Acquisition and Preprocessing

Data were scanned and preprocessed with a similar protocol of our previous study (Lei et al., 2020). Using a 3.0 TeslaMR system (GE Healthcare, GE Asian Hub, Shanghai, China), the fMRI data were obtained with gradient echo-planar imaging, time repetition/time echo = 2000/30 ms; flip angle = 90°; field of view = 220 × 220 mm^2^; slice thickness = 3.2 mm. The scan lasted for approximately 8 min. Data preprocessing procedures were performed with Statistical Parametric Mapping (SPM12; http://www.fil.ion.ucl.ac.uk/spm) and Data Processing Assistant for Resting-State fMRI (DPARSF) (Yan and Zang, 2010). Briefly, data were corrected, normalized, and spatially smoothed. A linear trend subtraction and temporal filtering (0.01–0.08 Hz) were performed to reduce the effect of low-frequency drifts and high-frequency noise. The cerebrospinal fluid and white matter were then regressed out as covariates. The Automated Anatomical Labeling (AAL-116) template was used to partition the fMRI data into 116 regions of interest (ROIs) (Tzourio-Mazoyer et al., 2002). Since the first 10 volumes were discarded from the total 240 time-points, the mean time series containing 230 volumes of each region was obtained by averaging the voxels within the region.

### Construction of FC Networks

**Figure 1:**
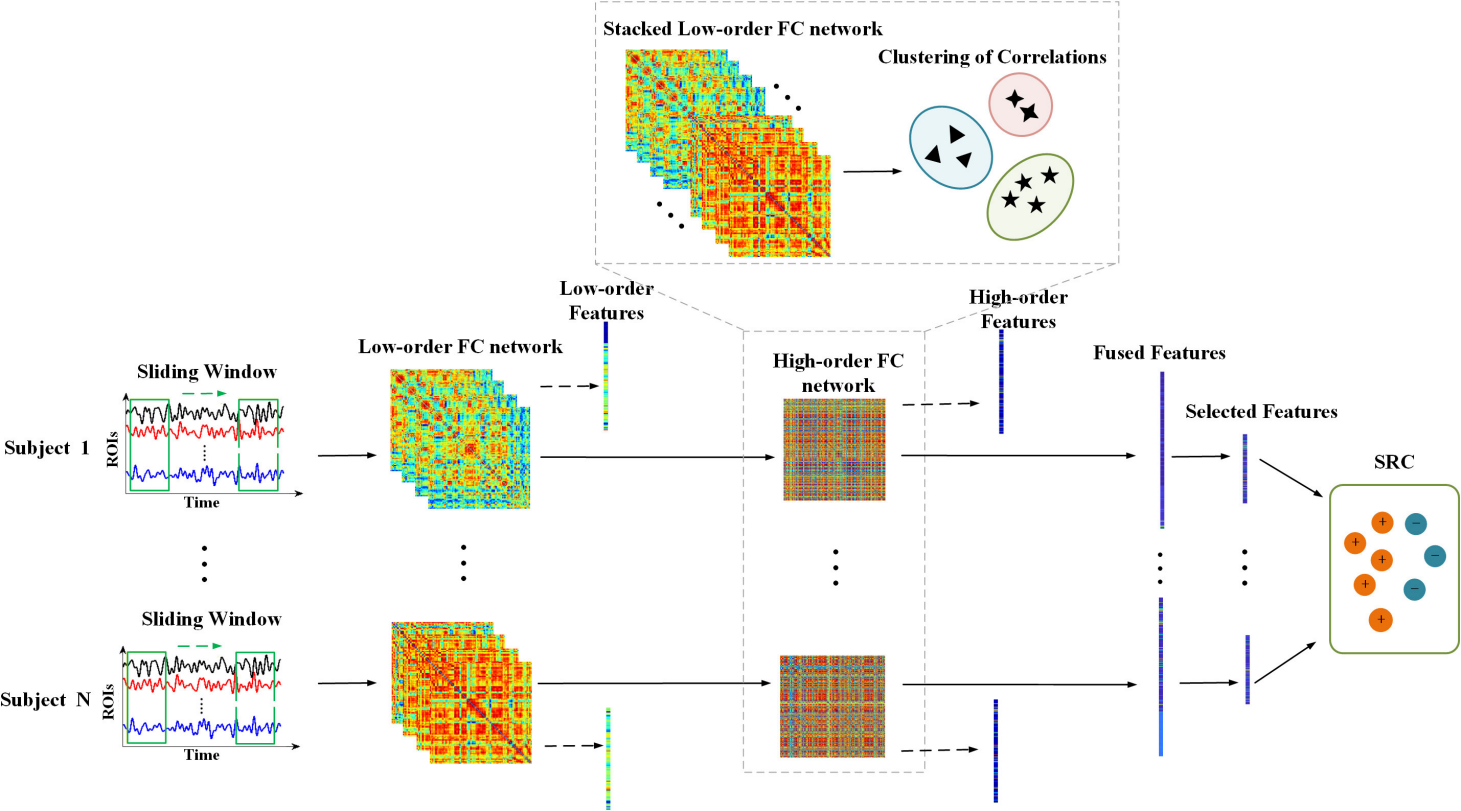
The overall framework of the proposed method. Procedure with dashed lines indicates the construction of high-order FC network. The dotted arrows mean extracting features from low-order and high-order FC networks, respectively, and the fused features consist of them.

Figure 1 shows the overall framework of the proposed method, and the dynamic low-order FC networks are constructed at first. Primarily, the entire time series of BOLD signal were partitioned into sub-series through the sliding window approach. Specifically, the time series with *T* temporal image volumes generates *K* = (*T* − *W*)/*S* + 1 sub-series, where *W* denotes the length of sliding window and *S* is the step size. Provided that $x_{i}^{\left(k\right)}\left(n\right)\in R^{W}$ is the *k-*th sub-series in the *i-*th ROI of the *n-*th subject, then $X^{\left(k\right)}\left(n\right)=\left[x_{1}^{\left(k\right)}\left(n\right),x_{2}^{\left(k\right)}\left(n\right),\ldots ,x_{Q}^{\left(k\right)}\left(n\right)\right]\in R^{W\times Q}$ represents the *k-*th sub-series in total *Q* brain regions of the *n-*th subject, *k* = 1, 2, …, *K*. A symmetric connectivity matrix $C^{\left(k\right)}\left(n\right)=\left[c_{ij}^{\left(k\right)}\left(n\right)\right]\in R^{Q\times Q}$, namely a low-order FC network, can be constructed using *X*^(*k*)^(*n*), where each entry in the matrix defines the Pearson's correlation strength between two different ROIs, that is:

$$\begin{array}{l} c_{ij}^{\left(k\right)}\left(n\right)=corr\left(x_{i}^{\left(k\right)}\left(n\right),x_{j}^{\left(k\right)}\left(n\right)\;\right) \end{array}$$

Let $\left\{x_{i}^{\left(k\right)}\left(n\right)\right\}$ be the nodes and their correlation strength $\left\{c_{ij}^{\left(k\right)}\left(n\right)\right\}$ stands for the weight of links between each pair of nodes, then *K* dynamic low-order FC networks can be estimated for *n-*th subject. Similarly, a static low-order FC network is the special case with *K* = 1. In this study, the length of sliding window *W* and the step size *S* were 90 and 1, respectively.

Afterwards, the dynamic high-order FC networks were generated through correlation of low-order FC networks layer by layer. As the correlation of correlations, a high-order FC network reveals more complex interaction relationships among brain regions. Firstly, a correlation time series of *n-*th subject can be expressed as $y_{ij}\left(n\right)={\left[c_{ij}^{\left(1\right)}\left(n\right),c_{ij}^{\left(2\right)}\left(n\right),\ldots ,c_{ij}^{\left(K\right)}\left(n\right)\right]}^{T}\in R^{K}$, which takes out the *i-j-*th elements from *K* low-order FC networks to record the variation of correlation between *i-*th and *j-*th ROI. And the high-order correlation of *n-*th subject is calculated as:

$$\begin{array}{l} h_{ij,rl}\left(n\right)=corr\left(y_{ij}\left(n\right),y_{rl}\left(n\;\right)\right) \end{array}$$

Since there are *Q*^2^ entries in a low-order FC network, which results in the number of {*y*_*ij*_(*n*)} being *Q*^2^, a high-order FC network will be $H\left(n\right)=\left[h_{ij,rl}\left(n\right)\right]\in R^{Q^{2}\times Q^{2}}$. Such large-scale network may not only lead to time consuming computation, but also trigger the curse of dimensionality when extracting local features from the network. Thus, a clustering method to group correlation time series was used to achieve network reduction (Chen et al., 2016).

In particular, we stacked all the {*y*_*ij*_(*n*)} of the total *N* subjects together to form a new item $Y_{ij}=\left[y_{ij}\left(1\right),y_{ij}\left(2\right),\ldots ,y_{ij}\left(N\right)\right]\in R^{K\times N}$ first. Then a hierarchical clustering method with minimum variance algorithm was implemented to generate *V* clusters that members in the same cluster share the similar variation in temporal dimension. Suppose that φ_*v*_ is the *v-*th cluster and |φ_*v*_| denotes the size of it, the mean correlation time series of the *v-*th cluster for the *n-*th subject is as follows:

$$\begin{array}{l} \bar{y_{v}}\left(n\right)=\frac{\sum_{ij\in \varphi_{v}}y_{ij}\left(n\right)}{\left|\varphi_{v}\;\right|} \end{array}$$

After that, a final high-order FC network after network reduction is constructed by regarding $\left\{\bar{y_{v}}\left(n\right)\right\}$ as the nodes and their correlation strength $\left\{\bar{h_{uv}}\left(n\right)\right\}$ as the weight of links, where $\bar{h_{uv}}\left(n\right)$ can be defined as:

$$\begin{array}{l} \bar{h_{uv}}\left(n\right)=corr\left(\bar{y_{v}}\left(n\right),\bar{y_{u}}\left(n\right)\right) \end{array}$$

### Feature Extraction and Selection

After the construction of low-/high-order FC networks, two weighted undirected graphs for each subject were generated. It is noteworthy that even if each subject has *K* temporal low-order FC networks, the final low-order FC network used in feature extraction is their averaged FC network. The local clustering coefficient, which was adopted to testify that the node's neighbors were still neighbors of each other, was calculated for feature extraction from these networks (Rubinov and Sporns, 2010). It indicates the prevalence of clustered connectivity around individual nodes and its specific definition is as follows:

$$f_{i}=\frac{\sum_{j\in N_{i}}{\left(w_{ij}\right)}^{\frac{1}{3}}}{\frac{1}{2}\left|N_{i}\right|(\left|N_{i}\right|-\;1)}$$

where *N*_*i*_ denotes the neighbors of node *i* and *w*_*ij*_ is the weight of link between node *i* and *j*. The denominator in the formula is the total number of possible links among node *i'*s neighbors.

Thus, concatenated feature sets including Q and V features from both low-order and high-order networks were generated for each subject. However, not all features are significant in classification due to their latent correlations. Besides, excessive features may lead to over-fitting. Therefore, a sparse representation (SR) and Locality Preserving Projection (LPP)-combined feature selection method was adopted to reduce some redundant features (Wu et al., 2019). The model is formulated as:

$$\begin{array}{l} \hat{w}=\arg\;min_{w}{\left\|y-w^{T}X\right\|}_{F}^{2}+\lambda_{1}tr\;\left(w^{T}XLX^{T}w\right)\lambda_{2}||w\;|{|}_{2,1} \end{array}$$

where *y* denotes the gold labels and *X* denotes the sample feature set. The *L* is the *Laplacian graph* and λ_2_ corresponds to a regularization item. Once the representation coefficient ŵ is calculated, the importance of features can be obtained by ranking the absolute value of each element in ŵ. Specifically, the higher the absolute value is, the more important the feature is. Then, a sequential forward selection algorithm is used to decide the ultimate feature subset for classification. In this algorithm, ranked features are added to the feature subset one by one, followed by classifier training and validation, then subsets with the highest classification performance are selected as the final result.

### Sparse Representation-Based Classification

There are two main steps of the SRC, computing representation coefficients and minimizing residuals between testing samples and their estimated results (Wright et al., 2009). For each class *i*, let δ_*i*_ be the characteristic function that selects the coefficients associated with the *i*-th class, then the following formulas show the corresponding steps mentioned above:

$$\begin{array}{l} \;\hat{w}=\arg\;min_{w}||w\;|{|}_{1}\;s.t.\;Xw\;=\sim y\\ identity(y)\;=\arg\;min_{i}\;r_{i}\left(y\right)=\arg\;min_{i}{\left\|y-X\delta_{i}(\hat{w})\right\|}_{2} \end{array}$$

where *X* is the whole training set and *y* is a test sample.

### Classifier Training, Validation, and Evaluation

A ten-fold cross-validation strategy was adopted because of the small sample size. The whole dataset was divided into 10 subsets, among which, nine-folds were used for training to find the optimal hyper-parameters, while the rest fold was used to test classification accuracy. Such procedure was repeated 10 times and the averaged results of these 10 rounds was deemed the final classification result. To evaluate the performance of the proposed method, metrics of the area under the receiver operating characteristic curve (AUC), accuracy (ACC), sensitivity (SEN), and specificity (SPE) were calculated.

### Algorithm Implementation

Incorporating the aforementioned classification and training strategies, details about algorithm implementation is shown in Supplementary Table 1. And evaluation metrics can be calculated using the predicted identity and its corresponding gold labels. All of the experiments were performed in Matlab R2015b.

## Results

### Participants

In experiment 1, all 79 patients with MMD were collected as positive samples for diagnosis modeling, and the 26 healthy subjects were used as negative samples. In the following experiment 2, the 36 patients with VCI were selected as positive samples, while the rest 43 patients with Non-VCI were used as negative samples. Detailed comparison of their demographic and cognitive features are showed in Table 1.

**Table 1:** Detailed demographics and cognitive testing of the subjects.

**Variables**	**VCI (*n* = 36)**	**Non-VCI (*n* = 43)**	**NC (*n* = 26)**	***F/X^**2**^* value (*p*-value)**
Age (years)	43.89 ± 10.38	38.51 ± 10.57	41.27 ± 11.15	2.51 (0.0866)
Male (%)	16 (44.44)	20 (46.51)	12 (46.15)	0.0364 (0.982)
Education (years)	8.58 ± 4.06	9.86 ± 3.86	8.96 ± 4.28	2.484 (0.2888)^a^
MMSE	21.14 ± 4.21	27.86 ± 1.52	28.23 ± 1.68	66.338 (0.001)^a^

*MMSE, Mini-Mental State Examination*.

a *Kruskal-Wallis test by ranks*.

### Low-Order FC Network Construction

**Figure 2:**
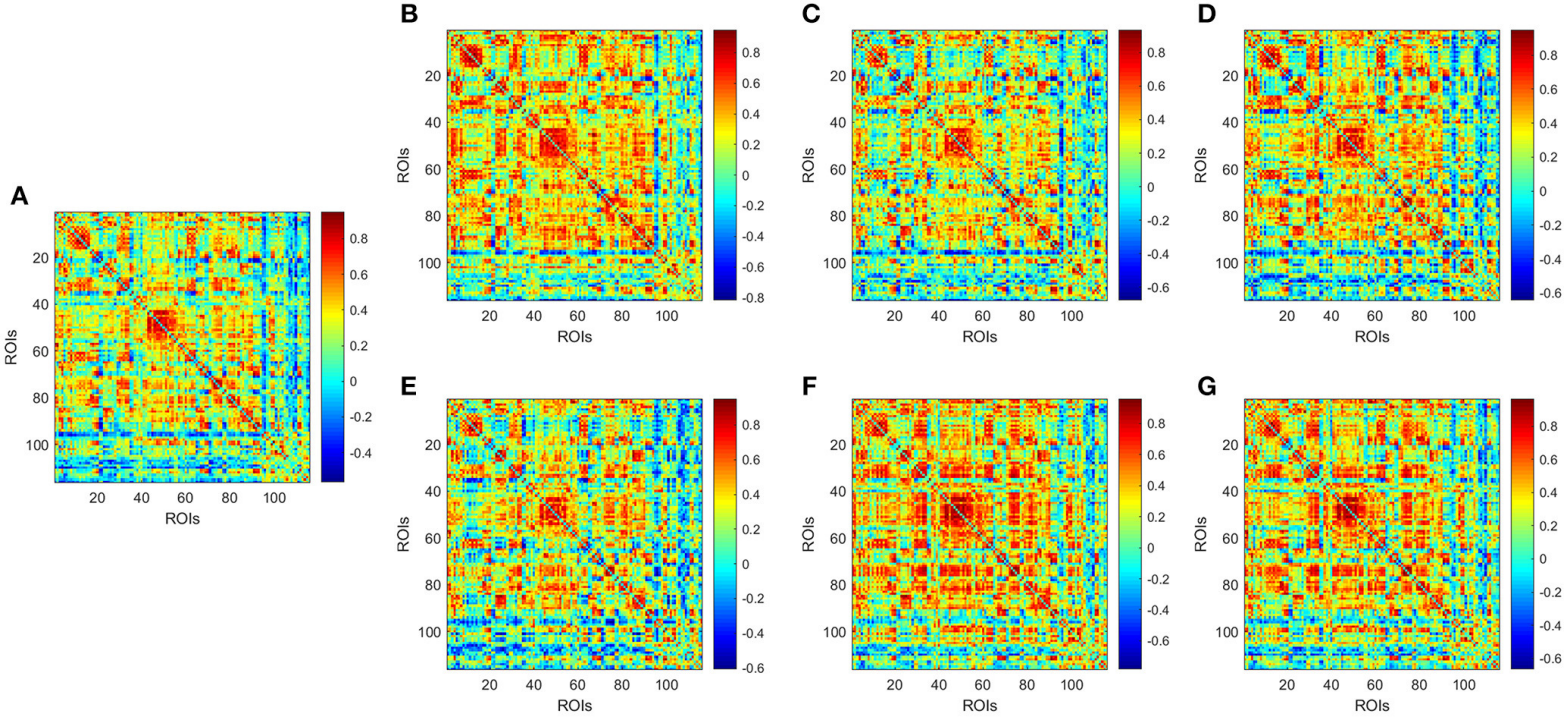
Low-order FC network for a random patient with MMD. **(A)** Shows the static network constructed by covering the whole-time volumes; **(B–G)** Show several dynamic networks in different sliding windows. Each element in the matrix is the correlation between two brain regions through the pairwise Pearson's correlation analysis. Element with light color indicates positive correlation, while the dark color shows a competitive or anti-correlation relationship between regions.

Figure 2 illustrates the temporal variations of BOLD signal by comparing a static low-order FC network covering the whole-time volumes with several dynamic FC networks randomly selected in different sliding windows in a patient with MMD. Results indicate that in different sliding windows, the correlation patterns among ROIs are not consistent, implying that dynamic FC network analysis provides more temporal information than the static network.

**Figure 3:**
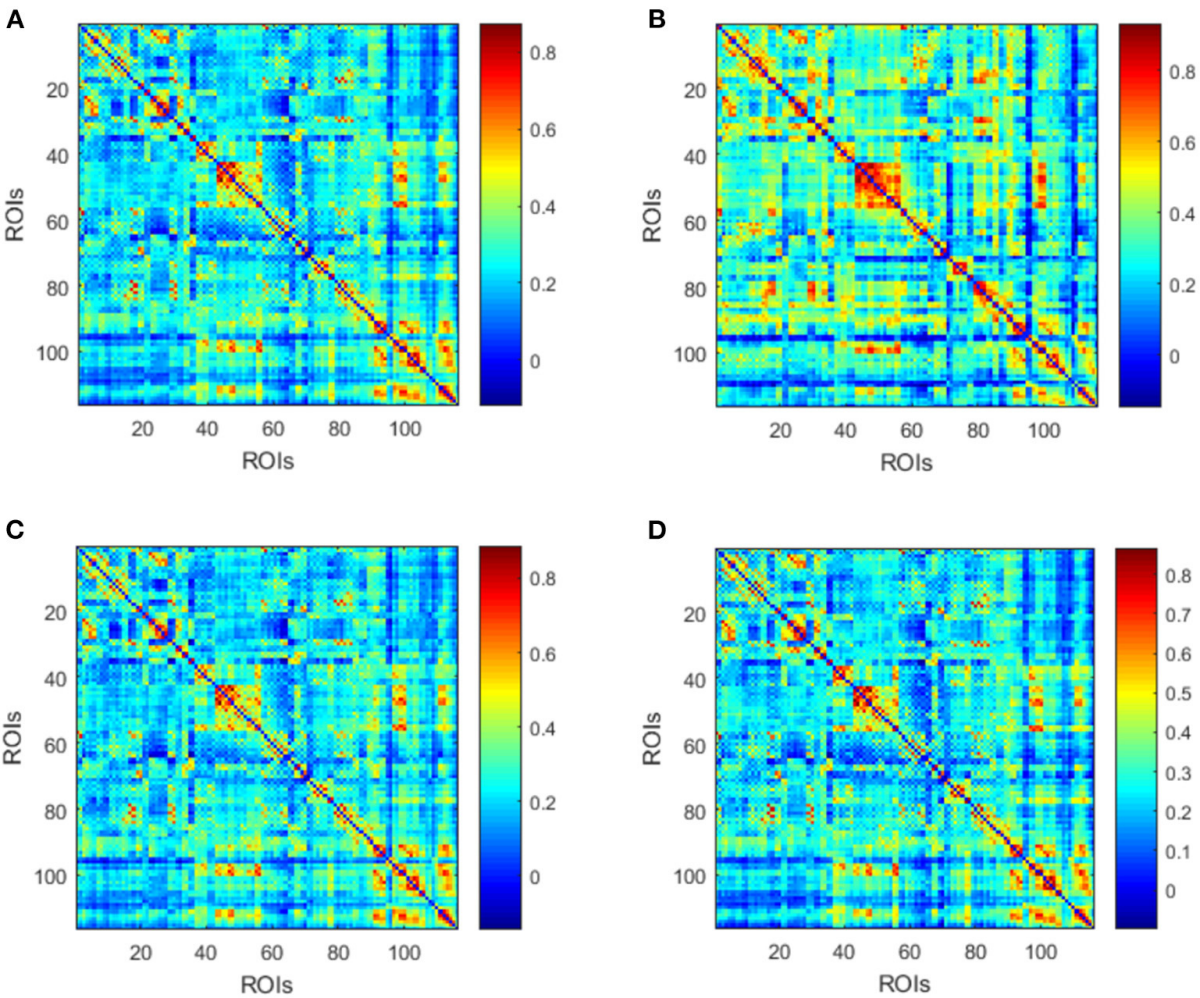
Averaged low-order FC networks for all MMD **(A)**, NC **(B)**, VCI **(C)**, and Non-VCI **(D)** groups, respectively. Each averaged network is generated by averaging networks of all sliding windows from all the corresponding subjects. Each element in the matrix is the correlation between two brain regions through the pairwise Pearson's correlation analysis. Element with light color indicates positive correlation, while the dark color shows a competitive or anti-correlation relationship between regions.

Afterwards, the averaged dynamic low-order FC networks in all siding windows from all the corresponding subjects were calculated and compared between the MMD and NC groups (Figures 3A,B). Visual inspection indicates that there are more correlations among ROIs in the NC group. Afterwards, similar comparison was performed between the VCI and Non-VCI groups, and generated less unremarkable difference (Figures 3C,D).

### High-Order FC Network Construction

For reducing computation complexity and other side-effect taken by reluctant features, a bottom-up hierarchical clustering method was adopted to divide correlated time series in all sliding windows from all subjects into some clusters. In the process, similar correlation information along the time series was included in the same cluster, and discriminative dynamic patterns were divided into different clusters.

**Figure 4:**
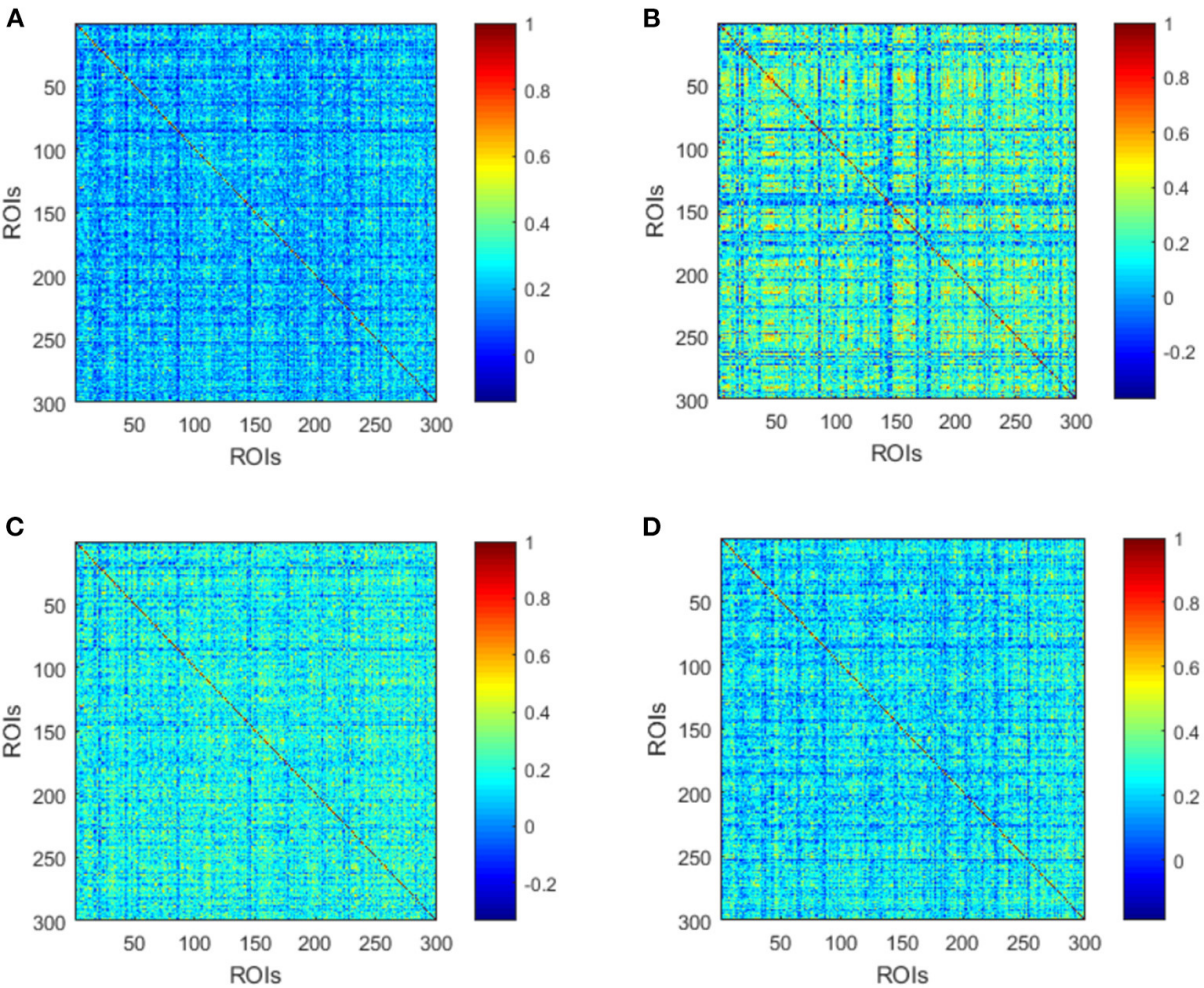
Averaged high-order FC networks for all MMD **(A)**, NC **(B)**, VCI **(C)**, and Non-VCI **(D)** groups, respectively. Each averaged network is generated by averaging networks of all sliding windows from all the corresponding subjects. Each element in the matrix is the correlation between two clusters through the pairwise Pearson's correlation analysis. Element with light color indicates positive correlation, while the dark color shows a competitive or anti-correlation relationship between clusters.

The averaged high-order FC networks in all siding windows from all the corresponding subjects were calculated and compared between the MMD and NC groups (Figures 4A,B). Although the two averaged networks still share similar patterns of correlation, the NC group exhibits more clusters with positive correlations. Next, unremarkable difference is noted when comparing averaged high-order FC networks of the VCI group with those of the Non-VCI group (Figures 4C,D). However, some latent and discriminative features can still be selected in the following part.

### Feature Selection

**Figure 5:**
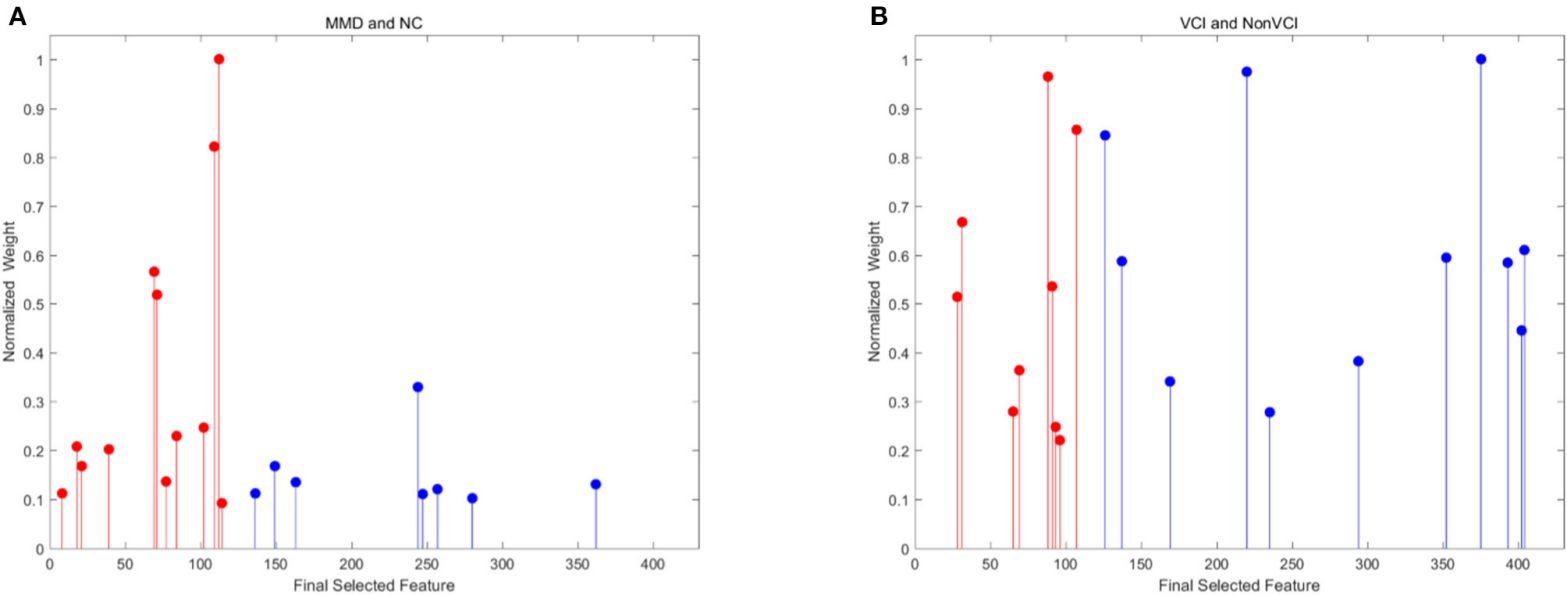
Normalized weight of final selected features for experiment 1 [**(A)** MMD and NC] and 2 [**(B)** VCI and Non-VCI]. These features are selected from 116 low-order features to 300 high-order features. The red color indicates low-order features, while the blue color represents high-order features.

**Figure 6:**
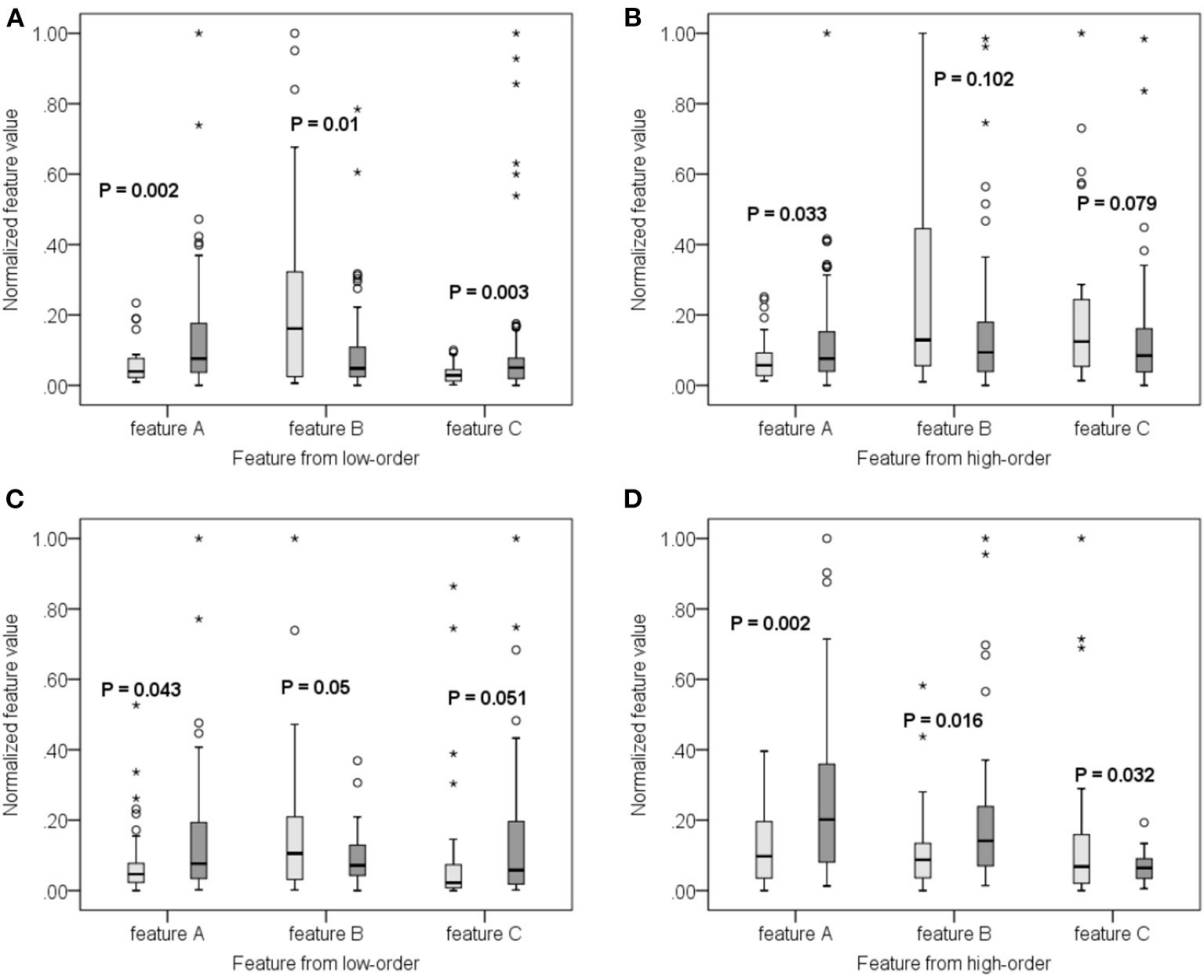
Boxplot of some features from both low-order **(A,C)** and high-order **(B,D)** networks for experiment 1 **(A,B)** and 2 **(C,D)**, respectively. The lighter color corresponds to negative samples while the darker color corresponds to positive samples.

Since the 116 time series of ROIs were generated from the low-order network and another 300 clusters were grouped from high-order network construction after investigating the effect of clustering number, a total of 416 features were extracted for each subject. Using the SR and LPP-combined method, followed by the sequential forward selection algorithm, 20 features were selected from experiment 1 and 2 as the most discriminative ones from the total 416 features. Their normalized weight was listed together and shown in Figure 5. In order to valid the discriminability of these selected features, we implemented the independent-sample *T*-test for each selected feature in two experiments and chose three features which had the minimum *p*-value from both low- and high-order sets to depict their boxplots (Experiment 1, Figures 6A,B; experiment 2, Figures 6C,D).

**Figure 7:**
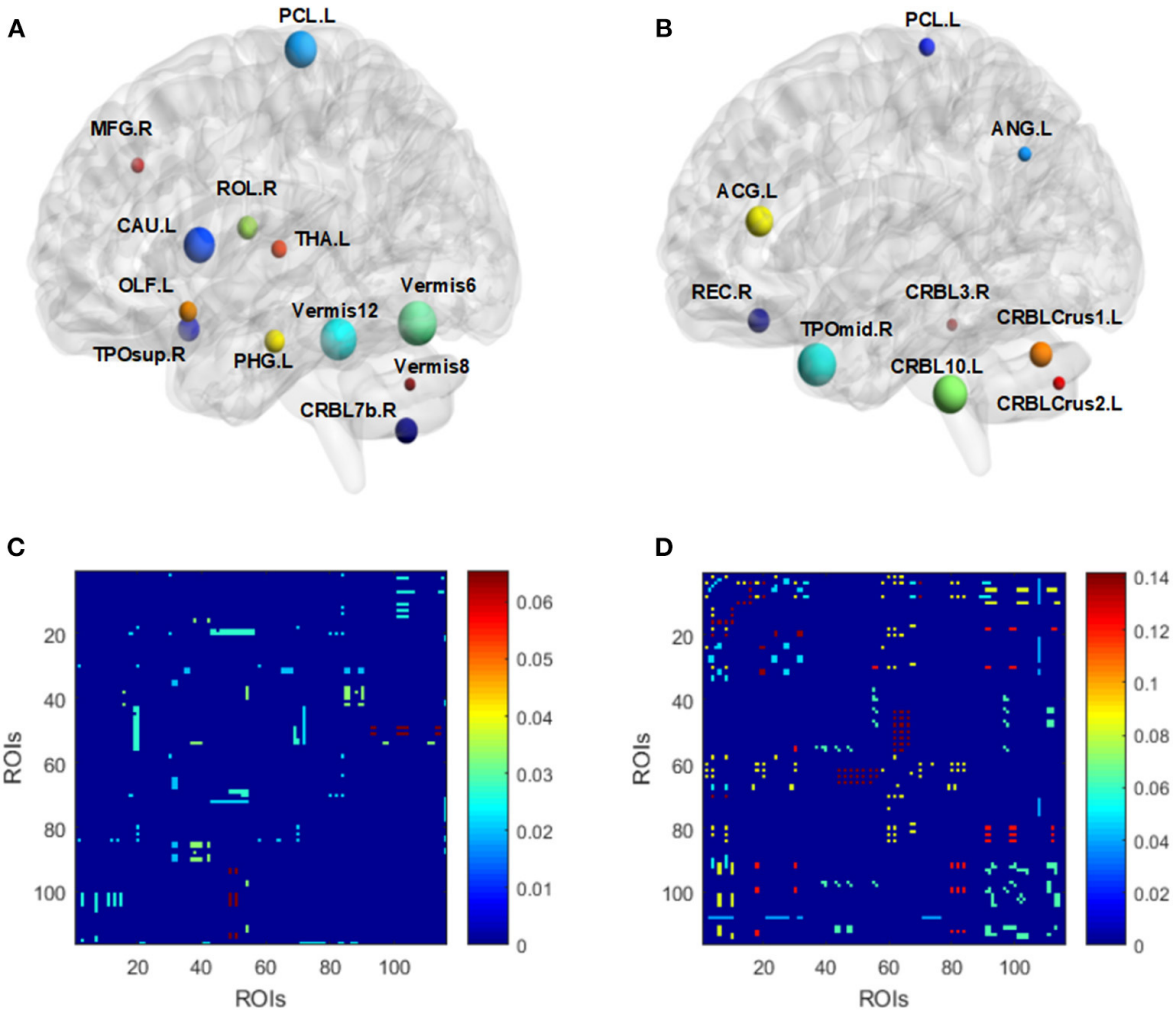
Visualized ROIs selected from both low-order **(A,B)** and high-order **(C,D)** networks for experiment 1 **(A,C)** and 2 **(B,D)**, respectively. For **(A)** and **(B)**, the size of nodes indicates the weight of corresponding ROIs. For **(C)** and **(D)**, the brighter the color is, the more important a cluster is **(C,D)**.

Referring to the low-order network, the corresponding brain regions from selected features in the two experiments are listed in Figure 7 and Table 2. Results indicate that feature patterns for experiment 1 and 2 are different. Referring to the high-order network, a cluster may consist of many ROI pairs instead of a certain brain region. Figures 7C,D Show the importance of selected clusters rather than ROI correlation matrices. The brighter the color is, the more important the cluster is.

**Table 2:** Features selected from the low-order FC networks.

**Experiment 1**		**Experiment 2**	
**ROI index^a^**	**Abbr**	**ROI index^a^**	**Abbr**
112	Vermis_6	88	Temporal_Pole_Mid_R
109	Vermis_1_2	107	Cerebelum_10_L
69	Paracentral_Lobule_L	31	Cingulum_Ant_L
71	Caudate_L	91	Cerebelum_Crus1_L
102	Cerebelum_7b_R	28	Rectus_R
84	Temporal_Pole_Sup_R	69	Paracentral_Lobule_L
18	Rolandic_Oper_R	65	Angular_L
39	ParaHippocampal_L	93	Cerebelum_Crus2_L
21	Olfactory_L	96	Cerebelum_3_R
77	Thalamus_L		
8	Frontal_Mid_R		
114	Vermis_8		

a *The Automated Anatomical Labeling (AAL-116) template*.

*Abbr, abbreviation; ROI, region of interest*.

### Classifier Validation and Evaluation

For experiment 1, the AUC, ACC, SEN, and SPE are 90.7, 88.57, 93.67, and 73.08%, respectively. Afterwards, those matrices for experiment 2 reach 91.02, 84.81, 80.56, and 88.37%, respectively.

## Discussion

In order to detect the VCI of adult patients with MMD in its early and reversible stage, the present study proposes a method to recognize the MMD (experiment 1) and its cognitive impairment (experiment 2) by using the low- and high-order features extracted from resting-state fMRI data. The promising classification results of two experiments with ten-fold cross-validation strategy clearly show that integrating time-varying properties in dynamic FC networks from different orders has great clinical significance.

The MMD is commonly reported to be accompanied with VCI, and surgical revascularization is proved to be beneficial at its early stage through improving cerebral hemodynamics (Gorelick et al., 2011; Karzmark et al., 2012; Lei et al., 2017a; Kazumata et al., 2019). Thus, timely and accurate detection of VCI is with clinical significance. Unlike neurodegenerative diseases such as Alzheimer's disease and Parkinson's disease, patients with VCI are normally with neurological defects such as hemiplegia and aphasia, and not suitable for regular neuropsychological testing. Besides, it is not easy to distinguish between mild VCI and normal cognition. Therefore, alternative measures with high accuracy and sensitivity should be developed. Nowadays, the resting-state fMRI is widely used to visualize brain functioning in both healthy and diseased subjects by relating to cognitive outcomes (Liang et al., 2016; Lei et al., 2017b). Specific to adult MMD, functional deficits of both regional neurons and global interactions are detected by comparing fMRI data of patients with healthy controls (Lei et al., 2014, 2017b; Kazumata et al., 2019). However, how to individualize and stabilize such measurement has become the next problem.

For recognition of VCI in adult MMD, dynamic low-order FC networks were primarily constructed and several features were extracted with high discriminability. Among these features, the anterior middle temporal gyrus is reported to be primarily involved in the default mode network (DMN), semantic retrieval, and sound recognition (Xu et al., 2015). The anterior cingulate cortex is a key node of salience network (Seeley et al., 2007). The gyrus rectus and left angular gyrus are both key nodes of DMN and reported to be involved in the brain reward response (Shott et al., 2015) and the retrieval of episodic and semantic information (Bonnici et al., 2016), respectively. Referring to the rest 4 features of cerebellum, they are also closely related to cognitive function (Gatti et al., 2020; Mannarelli et al., 2020; Van Overwalle et al., 2020).

Feature extraction from high-order FC networks is one of the most important reasons for outstanding recognition performance. After constructing dynamic low-order FC networks which indicates the correlation between two brain regions in a time-varying way, we assume that different pairs of brain regions could also influence each other, and their high-order correlation could contain more useful information for diagnosis. Thus, the Ward's linkage clustering is used for constructing high-order FC network, aiming at both mining the deeper interaction information and avoiding the curse of dimensionality (Chen et al., 2016). Compared with the low-order model, the high-order model discovers the dominant dynamic pattern from all correlation time series by clustering and obtaining more complex interaction relationships among ROI pairs. Besides, high-order networks are invariant to the chronological order of temporal low-order networks, and contribute to more consistent and meaningful comparisons across subjects. In the present study, we find out that there are more visual differences between healthy subjects and patients with MMD when comparing both the averaged low- and high-order networks. Besides, among the final selected features in experiment 2, high-order features present not only higher weight but also greater numbers, suggesting outstanding discriminability of high-order FC networks.

In most medical image researches, large number of features but small sample sizes are key issues which may lead to unsatisfactory results. To address this problem, some sparse representation (SR) methods were proposed in terms of feature selection and classification. For example, Lin et al. (2014) and Cao et al. (2014) used the SR for both fMRI and single nucleotide polymorphisms features selection in schizophrenia, while Yuan and Yan (2012) and Zhang et al. (2011) applied the SR into the final classification. In our study, two SR-related strategies are adopted which includes a SR and LPP-combined method for feature selection, and a SR-based classifier. The promising final recognition results validate the effectiveness of this proposed strategy. For feature selection, the SR regression is used to select discriminative features by giving inter-class the largest variance while intra-class the lowest variance. And the LPP is used to preserve the neighborhood structure of high-dimensional features even if they are projected into a new low-dimensional space, and improve the selection result by keeping the structural information. For classification, the SRC regards the test data as a combination of training data, and effective sparse coding will boost the final classification (Zhang et al., 2011). In addition, since medical images are associated with different kinds of uncertainty such as noise, low contrast, inadequate brightness, and so on, the SRC has advantages in handling such errors (Ghasemi et al., 2020).

Some limitations of this study should be addressed. Primarily, the generalization ability of the proposed method may be limited by the small sample size even though some SR techniques have been utilized to avoid over-fitting. Next, the sliding window method in constructing low-order network is a common and easy-implement way to generate dynamic FC networks. However, it simply divides the whole time series into several sub-series with manually-set window length instead of those special transition points obtained by exploring the intrinsic fluctuations. And such method may lead to confusing or less discriminative changes among different sub-series. Future studies are needed with larger dataset and more engineering strategies for improvement.

## Data Availability Statement

The raw data supporting the conclusions of this article will be made available by the authors, without undue reservation.

## Ethics Statement

The studies involving human participants were reviewed and approved by Institutional Review Board in Huashan Hospital. The patients/participants provided their written informed consent to participate in this study.

## Author Contributions

All authors listed have made a substantial, direct and intellectual contribution to the work, and approved it for publication.

## Conflict of Interest

The authors declare that the research was conducted in the absence of any commercial or financial relationships that could be construed as a potential conflict of interest.
