# Supplementary material for: Recognition of Cognitive Impairment in Adult Moyamoya Disease: A Classifier Based on High-Order Resting-State Functional Connectivity Network
Source: Front Neural Circuits. 2020 Dec 21;14:603208. doi: 10.3389/fncir.2020.603208 (PMC7779761; doi:10.3389/fncir.2020.603208)
Supplement: Supplementary file 1 [file Table_1.DOCX]

***Supplementary Material***

**Recognition of adult moyamoya disease and its cognitive impairment: a classifier based on high-order resting-state functional connectivity network**

Yu Lei^1^, Xi Chen^2^, Jiabin Su^1^, Xin Zhang^1^, Heng Yang^1^, Xinjie Gao^1^, Wei Ni^1^, Liang Chen^1^, Jinhua Yu^2*^, Yuxiang Gu^1*^, Ying Mao^1^

^1^Department of Neurosurgery, Huashan Hospital, Fudan University, Shanghai 200040, China

^2^Department of Electronic Engineering, Fudan University, Shanghai 200040, China

**Table S1 Algorithm implementation with SRC and ten-fold cross-validation**

| 1. Divide the whole dataset into ten folds equally, $k \leftarrow1$ |
| --- |
| 1. **repeat** |
| 1. $y\leftarrow k_{th} fold for testing , X\leftarrow the rest nine folds for training$ |
| 1. Obtain $\hat{w}$ from $\hat{w}=\arg{min}_{w} \left\Vert w \right\Vert_{1} s.t. Xw=y$ |
| 1. Obtain ${ID}_{k}(y)$ from ${ID}_{k}\left( y \right)=\arg{min}_{i} \left\Vert y-X\delta_{i}\left( \hat{w} \right) \right\Vert_{2} , i=1,2$ |
| 1. $k \leftarrow k+1$ |
| 1. **until** $k=10$ |
| 1. **return** ${\{ID}_{k}(y)\}, k=1,2,\ldots,10$ |
